# Developing a novel peer support intervention to promote resilience after stroke

**DOI:** 10.1111/hsc.12336

**Published:** 2016-03-04

**Authors:** Euan Sadler, Sophie Sarre, Anthea Tinker, Ajay Bhalla, Christopher McKevitt

**Affiliations:** ^1^ Division of Health & Social Care Research Faculty of Life Sciences & Medicine King's College London London UK; ^2^ National Institute for Health Research (NIHR) Collaboration for Leadership in Applied Health Research and Care South London Stroke Theme 5th Floor Addison House Guy's London UK; ^3^ NIHR Biomedical Research Centre Guy's and St Thomas’ NHS Foundation Trust and King's College London London UK; ^4^ National Nursing Research Unit Florence Nightingale School of Nursing & Midwifery King's College London London UK; ^5^ Institute of Gerontology Department of Social Science, Health & Medicine King's College London London UK; ^6^ Guy's and St Thomas’ NHS Foundation Trust St Thomas Hospital London UK

**Keywords:** complex intervention, peer support, resilience, stroke

## Abstract

Stroke can lead to physical, mental and social long‐term consequences, with the incidence of stroke increasing with age. However, there is a lack of evidence of how to improve long‐term outcomes for people with stroke. Resilience, the ability to ‘bounce back’, flourish or thrive in the face of adversity improves mental health and quality of life in older adults. However, the role of resilience in adjustment after stroke has been little investigated. The purpose of this study is to report on the development and preliminary evaluation of a novel intervention to promote resilience after stroke. We applied the first two phases of the revised UK Medical Research Council (UKMRC) framework for the development and evaluation of complex interventions: intervention development (phase 1) and feasibility testing (phase 2). Methods involved reviewing existing evidence and theory, interviews with 22 older stroke survivors and 5 carers, and focus groups and interviews with 38 professionals to investigate their understandings of resilience and its role in adjustment after stroke. We used stakeholder consultation to co‐design the intervention and returned to the literature to develop its theoretical foundations. We developed a 6‐week group‐based peer support intervention to promote resilience after stroke. Theoretical mechanisms of peer support targeted were social learning, meaning‐making, helping others and social comparison. Preliminary evaluation with 11 older stroke survivors in a local community setting found that it was feasible to deliver the intervention, and acceptable to stroke survivors, peer facilitators, and professionals in stroke care and research. This study demonstrates the application of the revised UKMRC framework to systematically develop an empirically and theoretically robust intervention to promote resilience after stroke. A future randomised feasibility study is needed to determine whether a full trial is feasible with a larger sample and wider age range of people with stroke.

**What is known about this topic**

- Resilience improves mental health and quality of life in older adults, so has relevance to stroke, the incidence of which increases with age.
- Little is known about the role of resilience in adjustment after stroke.
- The structured promotion of resilience has the potential to improve psychosocial outcomes after stroke, yet this has not been examined.

**What this paper adds**

- Resilience plays a role in promoting adjustment after stroke.
- The application of the revised UK Medical Research Council (UKMRC) framework enabled the systematic development and preliminary evaluation of an empirically and theoretically robust intervention to promote resilience after stroke.

## Introduction

Stroke is a leading cause of adult morbidity and mortality worldwide and a major public health issue (WHO 2014). It is commonly experienced as a long‐term condition, with one‐third of stroke survivors experiencing depression and poor quality of life up to 10 years after stroke (Wolfe *et al*. 2011, Ayerbe *et al*. 2014). In the United Kingdom (UK), a national survey found that half of stroke survivors reported long‐term unmet clinical and social needs (McKevitt *et al*. 2011). However, there is a lack of evidence of how to improve long‐term outcomes for people with stroke (Knapp *et al*. 2000, National Audit Office 2010).

Resilience is defined as a process of ‘bouncing back’, flourishing or thriving in the face of adversity (Netuveli *et al*. 2008, Hildon *et al*. 2009), for example when coping and adapting to a long‐term condition, and entails both positive trait characteristics and the ability of individuals to access a range of resources (Hildon *et al*. 2008). It has been proposed that resilience mediates between adversity and psychosocial adjustment following illness, which may explain why some people ‘bounce back’ following an adverse event or do ‘better than expected’ in adverse circumstances (Luthar & Brown 2007, Windle 2011). Resilience improves mental health and quality of life in older adults (Nygren *et al*. 2005, Netuveli *et al*. 2008, Hildon *et al*. 2009), so has relevance to stroke, the incidence of which increases with age.

In policy terms, resilience is increasingly recognised as a protective factor for health (Marmot 2010). In the UK, the Department of Health has stated that resilience is an important aspect of well‐being and mental health (Department of Health 2011). An influential House of Lords report ‘Ready for Ageing’ has demonstrated the costs for people aged 65 and over with disabilities, including stroke, will increase from 2010 to 2030 by over 50% (Filkin 2013: 58). This is one reason why they recommended effective measures to manage long‐term conditions. The structured promotion of resilience has the potential to improve psychosocial outcomes after stroke, yet this has not been examined. The purpose of this study is to report on the development and preliminary evaluation of a novel intervention to promote resilience after stroke.

## Methods

**Figure 1:**
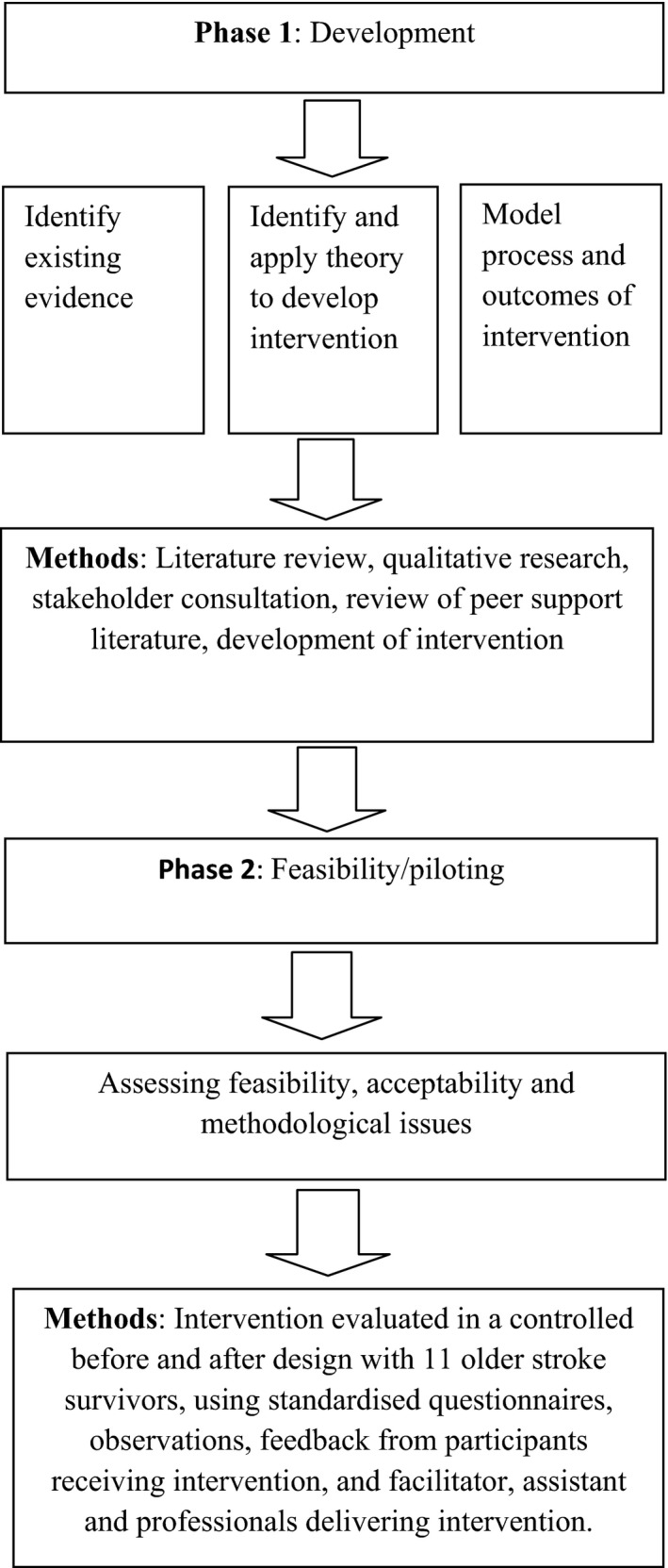
Process of intervention development and feasibility testing.

We used a mixed methods design guided by the revised UK Medical Research Council (UKMRC) framework for the development and evaluation of complex interventions (Craig *et al*. 2008). This framework defines a complex intervention as comprising a number of interacting components: different behaviours to deliver and receive the intervention, number of groups or organisational levels and range of outcomes (Craig *et al*. 2008: 979). It describes four cyclical phases: Development (phase 1): identifying existing evidence and theory, application of theory to develop the intervention, and modelling process and outcomes. Feasibility/piloting (phase 2): testing procedures for feasibility and acceptability. Evaluation (phase 3): understanding the process of change and assessing effectiveness and cost‐effectiveness. Implementation (phase 4): assessing and monitoring long‐term effectiveness and dissemination (Craig *et al*. 2008: 980). In this paper, we focus on phases 1 and 2 – development and assessment of feasibility of a complex intervention to promote resilience to improve psychosocial outcomes after stroke (see Figure 1).

### Data collection

First, we conducted a scoping review of the resilience literature to identify existing evidence and theory to inform intervention development. In light of a lack of evidence of studies examining resilience after stroke, to develop the theoretical foundation of the intervention we conducted: (i) a systematic review of qualitative studies on adjustment after stroke and (ii) qualitative research with older stroke survivors, carers and professionals to investigate understandings of resilience and its role in adjustment after stroke. In‐depth interviews were conducted between May and October 2012 with 22 stroke survivors aged 60 and over. Participants were recruited from the South London Stroke Register (SLSR), a population stroke register covering an ethnically diverse inner city region (Heuschmann *et al*. 2008). We used purposive sampling to recruit a diversity of participants across gender, ethnicity and level of disability. Three interviews were conducted jointly with family carers, while two interviews were undertaken separately with two further carers. Between October 2012 and March 2013, we also undertook focus groups and individual interviews with 38 professionals involved in the delivery and commissioning of stroke and older adult services. All interviews and focus groups were audio recorded with participant consent. Ethics approval for the qualitative work was granted by the National Research Ethics Service (NRES) Committee South West‐Central Bristol (REC reference number: 12/SW/0063).

### Qualitative analysis

Qualitative data were transcribed and imported in NVivo (Version 10). All transcripts were read in full and coded for themes. We undertook a systematic thematic analysis of codes and categories to develop and refine themes emerging from the raw data (Braun & Clarke 2006). A discussion then took place between three authors (ES, SS, CM), to reach a consensus on the main themes. Rigour was achieved by adopting a systematic and transparent approach to the analysis (Seale & Silverman 1997).

### Intervention development and evaluation

The findings were discussed with a stakeholder panel to agree the components of an intervention. Stakeholders included stroke researchers, health professionals and service user representatives from the King's College London Stroke Research Patients and Family Group. Findings were initially presented to stakeholders who were asked to consider in light of the evidence, the types of intervention that might be feasible. The larger group then reconvened for group discussion to co‐design an intervention. As a peer support model had been proposed, we scoped this literature to identify likely mechanisms of change that could operate as ‘active ingredients’ of peer support to improve resilient practices after stroke. We then modelled process and outcomes to develop a theoretically informed intervention to promote resilience after stroke. Finally, we conducted an initial feasibility study of the intervention in a community setting with older stroke survivors, for which ethics approval was granted by the NRES Committee North West‐Greater Manchester West (REC reference number: 13/NW/0627).

## Findings

### Identifying existing evidence and theory

We searched SCOPUS, Cochrane Database of Systematic Reviews and PsycInfo to identify existing evidence and theoretical mechanisms likely to explain the effectiveness of an intervention promoting resilience to improve psychosocial outcomes after stroke. We found a lack of interventions, so we searched for interventions targeting older people and those with other long‐term conditions. We found one study reporting a randomised controlled trial (RCT) of a 10‐module group ‘resiliency training’ intervention for people with diabetes (Bradshaw *et al*. 2007). The intervention group demonstrated higher levels of resilience, in terms of higher reported use of positive coping strategies, improved healthy lifestyle choices and increased physical activity levels at 3 months, compared to a control group (Bradshaw *et al*. 2007).

We then searched for observational studies examining resilience after stroke, which also revealed a lack of studies, so we looked for evidence of: (i) predictors/determinants of resilience in older people and those with long‐term conditions; and (ii) factors identified to promote resilience among older people (Table 1).

**Table 1:** Predictors/determinants of resilience in older people and those with long‐term conditions, and factors identified to promote resilience among older people

Predictors/determinants	Factors identified by older people
*Older people* Adaptive coping styles (Lamond *et al*. 2008, Hildon *et al*. 2009); lower levels of depression (Hardy *et al*. 2004); higher quality of social support (Netuveli *et al*. 2008, Hildon *et al*. 2009); higher levels of social participation (Lamond *et al*. 2008) and community integration (Hildon *et al*. 2009) *People with long‐term conditions (e.g. diabetes, cancer, rheumatoid arthritis, HIV)* Psychological factors (e.g. self‐efficacy, self‐esteem, determination); social support from family and friends; positive coping strategies to adapt to illness (e.g. cognitive appraisal, spirituality) (Stewart & Yuen 2011)	Positive personality trait characteristics and attitudes (Felten & Hall 2001, Windle *et al*. 2010, Wiles *et al*. 2012); quality of social support and lifelong coping strategies (Felten & Hall 2001, Hildon *et al*. 2008); opportunities to help others (Felten 2000, Felten & Hall 2001, Kinsel 2005); access to care (Felten 2000, Felten & Hall 2001); spiritual and religious resources (Felten 2000, Felten & Hall 2001, Clarke & Cordman 2002, Becker & Newsom 2005, Kinsel 2005)

Overall, findings showed that resilience comprises a range of psychological, social and environmental factors (Windle 2011). One multidimensional framework of resilience proposed in the literature is the ecological systems theory (Bronfenbrenner 1979, Ungar 2011). This model suggests that resilience operates on three levels: individual, interpersonal and structural levels, reflects a person's ability to access resources, and convert these into good outcomes.

### Developing the theoretical foundation of the intervention

#### Review of qualitative studies on adjustment after stroke

In light of a lack of intervention studies examining resilience after stroke, we conducted a systematic review of qualitative studies of older stroke survivors’ experiences of adjustment after stroke. Full details of the methods have been published elsewhere (Sarre *et al*. 2014). Key findings informing intervention development were:

- Stroke survivors identified multiple factors promoting adjustment after stroke, operating on individual, social and organisational levels, which is largely in line with the ecological systems theory of resilience (Bronfenbrenner 1979, Ungar 2011).
- Stroke survivors reported practical and mental coping strategies to promote adjustment after stroke. The former included relearning new tasks and goal setting, whereas the latter involved drawing on meaning frameworks and making comparisons with other stroke survivors perceived as being ‘worse off’.
- Social support (i.e. practical, emotional, moral support) provided by family and friends and peer support groups were identified by stroke survivors as promoting adjustment after stroke.
- Organisational factors hindering adjustment after stroke included lack of access to health and social care and lack of information tailored to meet individual needs.

#### Stroke survivor and carer understandings of resilience after stroke

To identify stroke survivor understandings of resilience and its role in adjustment after stroke, we conducted interviews with 22 individuals, aged 62–89 years, between 8 and 22 months post stroke. Thirteen participants were white British, six black Caribbean and three black African; seven were female and 15 male. Five interviews (three joint and two separate) were conducted with spouse carers. In examples of supporting data extracts, S01F/M, S02F/M refers to stroke survivor 1, 2 (female/male) and so on, whereas C01, C02 refers to carer 1, 2 and so on.

Lay understandings of resilience commonly reflected positive personal characteristics, personality traits or attitudes of the individual in relation to one's recovery following a stroke. This included having a sense of personal endurance to overcome the stroke:

Somebody who's able to unpack within a situation what is constructive and try and bypass what's not constructive … now it's either going to get me or I'm going to get it. (S11F)

It's like sustainability isn't it?‐durability. (S05M)

Several participants viewed resilience as encompassing ideas related to agency, coping with ‘setbacks’ and withstanding stress. This reflected having ‘a fighting spirit’ and the ability to ‘bounce back’, ‘keep going’, ‘adapt’ and ‘be resourceful’ in the face of coping with illness and other adversities in life:

Having a fighting spirit. I don't give in to anything. (S01F)

I'm able to recover from setbacks. Well I suppose just in‐built really isn't it? Some people have that facility and some don't. (S07F)

Someone who is resilient they have this capacity of bouncing back. (S13M)

Resilience was further defined by a minority of women as a lifelong trait shaped by one's upbringing: ‘It's the way I've been brought up’ (S10F). It also reflected the ability of an individual to overcome earlier adverse life hardships, such as poverty or experiencing the Second World War.

Older stroke survivors largely reported similar practical and mental coping strategies that we found in our review. In addition, some participants adopted the mental strategy of ‘downplaying’ the impact of the stroke by making light of it. Several participants viewed resilience as both a trait and a social process, drawing on resources and activities through social participation in community groups. For example, one man said:

[It's] about strength, determination … I think also an element of it, of this resourcefulness, is doing things like signing up for yoga, that sort of thing, accessing help or things that help you. (S20M)

Similar to our review, participants made social comparisons with others perceived to be ‘worse off’ as part of adjusting to the physical effects of their stroke:

Well, I look, even that, those people who I see have lost their limbs, and when I see [they] are still living on, trying in life, doing things, I thought to myself well, I'm not the worst, I'm lucky too. (S19M)

Social support from family and friends was commonly identified among participants as an important component of adjusting to life after stroke. This included practical support, for example, shopping, cleaning and driving. Other types of support included help with accessing information, home adaptations and sheltered housing. Family members were also viewed as a source of moral support and encouragement, helping some to cope with isolation following the stroke:

From the beginning when you are alone, you find it difficult without the help. But with the help and encouragement you find it easy … but with their [family] help and advice you can do it, then you overcome it. (S14M)

Peer support groups were identified as a resource to foster resilience and adjustment following the stroke for a number of participants. This was through mechanisms such as learning from other people coping with the impact and consequences of a stroke, opportunities to share experiences and role model behaviours:

When I go to this gathering [a stroke club] it's the interaction with people, discussing with people who have similar problems. You can see how they manage, to follow suit. (S16M)

Compared to our review, participants more commonly reported organisational factors limiting their ability to access services, which they considered would promote recovery and adjustment following the stroke. This included lack of access to rehabilitation services and home adaptations to improve independence and social participation, and services to support coping with the emotional and psychological consequences of the stroke.

Carers of stroke survivors largely reported similar shared understandings of resilience. In particular, they spoke about the importance of positive personal characteristics and having flexible coping strategies to adapt to the consequences of the stroke:

Life is not over. Adapt yourself and keep on because you can do things. (C04)

You're not going to be batted back … I think it's a sort of stubbornness, determination that you are going to find a way … If you think well I've done my best shot at it, it didn't work so I'm going to go and do something completely different, maybe that's a better result. (C05)

In sum, resilience resources identified by older stroke survivors reflected multiple domains, comprising individual, organisational, social and structural aspects, further lending support for the ecological systems ecological systems theory (Bronfenbrenner 1979, Ungar 2011).

#### Professional understandings of resilience

Thirty‐eight professionals took part in group (*N* = 34) or individual interviews (*N* = 4) to investigate their understandings of resilience and its role in adjustment after stroke. Participants included health professionals working in hospital (e.g. physiotherapists [HP] and community stroke rehabilitation (e.g. community occupational therapists [COT], a service manager for a charity for people with aphasia [SMA], a commissioner of older people's services and a local stroke care advisor [SCA].

Professional understandings of resilience similarly commonly reflected positive personal characteristics and personality traits. These were considered to influence a person's attitude, motivation and response to adapting to the long‐term consequences of stroke:

It's more related to personality than anything else. Some people just do have a much more positive view on things and they will engage more with what's offered. (SCA)

Resilience was further viewed among health professionals as a person's ability to mentally adapt, cope and ‘bounce back’ following a stroke. It was also related to positively coping with other setbacks more generally in life. For example, one community physiotherapist understood resilience as:

The ability to bounce back, so if you have a setback, it's your ability to get yourself back up.

Stroke professionals perceived that having access to a range of resources reinforced a person's resilience and adjustment after stroke. Resources included information tailored to meet individual needs, access to health and social care, and services providing psychological, emotional and social support, self‐management and peer support. For example, one hospital physiotherapist spoke about the positive effects of peer support in terms of providing opportunities to foster resilience through social comparison with other stroke survivors:

I think a lot of peer support as well … it's all about seeing someone who's in a worse situation than you doing better and it's always thinking I'm not the worst off, there are people in worse situations than me and look what they've achieved. (HP)

Resilience was not only viewed by stroke professionals and care providers in terms of individual trait characteristics but also social practices. The quality of social support provided by family and friends was perceived as a key component influencing long‐term adjustment after stroke, and a potentially valuable resilience resource:

Resilience is something that you have to practice as a family … to bolster one person's resilience can have a ripple effect. (SMA)

A good social network, that's number 1. Not being isolated and alone because if there are people around them all getting on with life and they feel as if they're part of something. (COT)

Service providers, like some stroke survivors, also reported organisational factors as barriers to promoting resilience and adjustment after stroke, in terms of a lack of existing services to support people to deal with emotional and psychological consequences after stroke. Some also spoke about the need for a ‘reablement culture’ and for services to be patient‐centred, and tailored to individual needs and goals.

In summary, we found similarities between how stroke survivors, carers and professionals understood resilience, reflecting mainly resilient trait characteristics and social practices.

#### Stakeholder consultation

We discussed our findings with a stakeholder group, comprising stroke researchers, health professionals and service users, to co‐design the intervention. This led to advice on the following characteristics of an intervention:

- Target multiple practices to promote resilience after stroke (meaning‐making through sharing experiences, management of long‐term physical health issues, fostering coping strategies, participation in activities and roles, accessing tailored information and services, and developing opportunities to help others);
- Group‐based intervention underpinned by a peer support model, defined as support between people who have an experience in common (Dennis 2003);
- Target population stroke survivors 6–24 months post stroke, identified as a key period of psychosocial adjustment after stroke (Kendall *et al*. 2007);
- Target group size up to 15 people, which was considered a manageable size based on our previous research experience;
- Facilitated by two stroke survivors for a group this size, acting as role models for the group;
- Written information to accompany the programme as memory problems are a common consequence of stroke (McKevitt *et al*. 2011);
- Outcome assessment to include mental health and quality of life, as these are positively associated with resilience in older adults (Nygren *et al*. 2005, Netuveli *et al*. 2008, Hildon *et al*. 2009).

#### Scoping the peer support literature

As preliminary work indicated that a peer support model would inform the intervention, we returned to the literature to: (i) assess evidence of peer support programmes for people with stroke and other long‐term conditions; and (ii) theoretical mechanisms likely to explain the effectiveness of a peer support group intervention promoting resilience to improve psychosocial outcomes after stroke.

A concept analysis of peer support identified three components of peer support: emotional, appraisal (e.g. encouragement, affirmation) and informational (Dennis 2003). We found one group‐based peer support intervention for younger stroke survivors (aged under 65 years) (Muller *et al*. 2014). The intervention was informed by theories including group dynamics theory (Jacobs *et al*. 2012) and social learning theory (Bandura 1977) (e.g. providing a sense of belonging, peer‐to‐peer learning, modelling behaviours, problem‐solving and goal setting), and led to improvements in socialisation, healthy coping and role attainment (Muller *et al*. 2014).

Outside stroke, peer support programmes for individuals with cancer (Campbell *et al*. 2004, Hoey *et al*. 2008) and heart disease (Parry & Watt‐Watson 2010) have found mixed evidence of psychosocial benefit. Methodological issues exist in terms of different types of peer support interventions evaluated (e.g. telephone, one‐to‐one, group approaches), with most lacking conceptual grounding. One exception was a 6‐week group intervention (supplemented by a workbook) delivered by two peer facilitators for people with heart or lung disease, or diabetes, underpinned by social learning theory (Bandura 1977), which significantly improved health behaviours, health status, and self‐efficacy, and reduced emergency visits at 4 and 12 months follow‐up (Lorig *et al*. 2003).

Qualitative studies further identify theoretical constructs of peer support that promote adjustment after stroke, including peer‐to‐peer learning, modelling of behaviours and social comparison (Kvigne *et al*. 2004, Ch'ng *et al*. 2008, Brown *et al*. 2010). Older people have been found to make downward social comparisons (to those they regard as ‘worse off’) to positively appraise their well‐being and quality of life in the face of ill‐health (Beaumont & Kenealy 2004). The response shift model, defined as a shift in a person's appraisal of a given life event, proposes changes in people's values towards a more outward looking orientation (Schwartz & Sendor 1999). In this model, helping others brings about a more outward looking orientation and is proposed to increase self‐esteem (Campbell *et al*. 2004). Finally, peer support provides opportunities for meaning‐making through sharing experiences with others, as part of adjusting to chronic illness (Hydén 1997, Greenhalgh 2006, Newbould *et al*. 2007).

### Modelling process and outcomes

Based on the existing evidence and theory, and findings from our research, we designed a 6‐week group‐based peer support intervention to promote resilience after stroke: ‘Back on Track’. The intervention targets a number of practices to enhance resilience after stroke. These are meaning‐making through sharing experiences; improving management of long‐term issues (e.g. physical health; managing medication regimes); fostering practical and mental coping strategies; providing support to identify and participate in valued social activities and roles; provision of information tailored to individual needs; facilitating access to local community services; and developing opportunities to help others. We proposed that the theoretical mechanisms of peer support likely to improve resilience and psychosocial outcomes after stroke would be through social learning, meaning‐making, helping others and social comparison (see Table 2).

**Table 2:** Developing the theoretical foundation of the ‘Back on Track’ intervention

Aim	Means	Mechanisms of change
To promote resilience after stroke	Enhance resilience practices by means of a group peer support intervention	Social learning, meaning‐making, helping others, social comparison
Objective	Means	Evidence of mechanisms of change
To provide an opportunity to give and receive emotional, appraisal, informational and instrumental support between peers.	Formal and informal exchange of information, knowledge and understanding between stroke survivors	Helping others increases self‐esteem in adults with cancer (Hoey *et al*. 2008). Downward social comparisons improves perceptions of well‐being in older adults (Beaumont & Kenealy 2004). Group‐based peer support interventions based on social learning theory (peer‐to‐peer learning, modelling behaviours and coping strategies, problem‐solving and goal setting) improves socialisation, healthy coping and role attainment in stroke survivors (Muller *et al*. 2004), and self‐efficacy, health behaviours and health status for people with heart or lung disease, or diabetes (Lorig *et al*. 2003).
To foster practical and mental coping strategies for managing life after stroke.		Older stroke survivors use a range of practical and mental coping strategies to promote resilience (our qualitative research) and adjustment after stroke (Sarre *et al*. 2014) Our qualitative research found that older stroke survivors identified peer support as a factor promoting resilience to aid adjustment after stroke (through peer‐to‐peer learning, sharing experiences, and modelling behaviours).
To provide an opportunity to make sense of stroke and its consequences in a social context.	Stroke survivors sharing experiences with others.	Meaning‐making through sharing experiences with others promotes adjustment to chronic illness (Hydén 1997, Greenhalgh 2006, Newbould *et al*. 2007). Older stroke survivors use meaning‐making strategies to promote adjustment after stroke (Sarre *et al*. 2014)
To help people to identify personally valued social activities and roles.	Facilitating reflection and problem‐solving; sharing information and experiences between peers.	Group‐based peer support interventions based on social learning theory (peer‐to‐peer learning, modelling behaviours and coping strategies, problem‐solving and goal setting) improves socialisation, healthy coping and role attainment in stroke survivors (Muller *et al*. 2004), and self‐efficacy, health behaviours and health status for people with heart or lung disease, or diabetes (Lorig *et al*. 2003)
To provide information tailored to individual needs.	Providing information on local services, practical support for relevant information, sharing of information between peers, and referral to local services.	Lack of information tailored to meet individual needs and lack of access to services hinders adjustment after stroke (Sarre *et al*. 2014) (and our qualitative research).

The intervention would be facilitated by two stroke survivors (one facilitator and one assistant) acting as role models for the group, having achieved a good level of adjustment and recovery following their stroke. The programme comprises two 50‐min‐long module sessions, running once a week for 6 weeks, including a mixture of group discussions and reflection activities, with invited input from a nurse, community stroke care advisors working in the local voluntary sector, stroke researchers and stroke survivor volunteers. Participants are provided with a workbook with an outline of the course and self‐completion ‘valued activities sheets’ providing them with the opportunity to record ideas and set goals.

### Assessing feasibility of the intervention

We conducted an initial feasibility study of the intervention with older stroke survivors in a local community setting to assess rates of recruitment and retention, rates of completion of outcomes and perceptions of acceptability. The intervention was evaluated in a controlled before and after design, using standardised questionnaires, observations of group module sessions, feedback forms and qualitative interviews with a subsample of participants taking part, and email feedback from peer facilitator, assistant and professionals delivering the intervention.

Stroke survivors were recruited from the SLSR. Inclusion criteria were: aged 60 years and over, 6–24 months post stroke, living in the community, and able to provide informed consent. A log was kept of rates of recruitment and retention to the intervention. Of 50 stroke survivors approached by telephone, we were unable to contact 10 people, while 25 declined to take part for a number of reasons (e.g. too busy, poor health, lack of perceived benefit). Fifteen people initially gave verbal consent, but of these 11 decided to take part in the intervention, representing a 22% response rate, including seven men and four women, age range 63–87 years; 6–23 months post stroke. Seven participants were white British, three black Caribbean and one black African. Attendance at module sessions was variable: six participants attended all module sessions; two attended five sessions, and one person each took part in three, two and one session respectively. About 15% of sessions were missed due to ill‐health, hospital appointments or reported fatigue. Nine of 11 participants were unable to use public transport because of stroke‐related disabilities, and would not have been able to take part in the programme without transport being provided.

Standardised questionnaires were used to measure baseline and post‐intervention outcomes at 6 weeks. The primary outcome examined resilience using the Brief Resilience Scale (Smith *et al*. 2008) which has been validated in adults with chronic illness (Windle *et al*. 2011) and has the advantage of being short (six items; 0 lowest and 6 highest resilience score). Secondary outcome measures examined level of activity, health‐related quality of life (HRQOL) and mental health. Level of activity was measured with the Frenchay Activities Index (range from 0 = inactive to 45 = active) which has been validated in stroke (Holbrook & Skilbeck 1983). HRQOL was assessed using the UK version of the Medical Outcomes Study 12‐item short form (SF12), which is widely used, and has good psychometric properties (Wolfe *et al*. 2011). HRQOL is divided into physical health QOL (PHQOL) and mental health QOL (MHQOL), each score ranging from 0 (lowest) to 100 (highest). Finally, mental health was measured using the Hospital Anxiety and Depression Scale (HADS) (Zigmond & Snaith 1983), which has also been validated for use with stroke survivors (Aben *et al*. 2002, Wolfe *et al*. 2011). HADS records level of anxiety and depression, each score ranging from 0 (lowest) to 21 (highest).

Of the 10 of 11 participants completing baseline and follow‐up outcome measures, results showed a marginal increase in mean resilience scores (3.6–3.8). Resilience scores varied within the group; six participants having slightly increased and four slightly decreased scores. The latter subgroup could be explained by three of these four participants reporting relatively high baseline scores. In addition, there was no change in mean activity levels (19.0–18.9) and depression scores (5.5–5.6); a marginal increase in mean PHQOL (32.9–34.4) and MHQOL (37.0–38.6); with a slight increase in mean anxiety scores (4.3–5.5). One explanation for these results is that time of follow‐up was short, highlighting the need for a longer follow‐up period in a future trial. A further issue was missing outcome data, particularly among participants with higher levels of physical disability, limiting their ability to read or write to complete the questionnaires. Missing data for follow‐up outcomes among several participants were also affected by memory problems following the stroke. In a future trial, flexibility needs to be given to providing support to such participants, by telephone or face‐to‐face, to complete outcomes.

Qualitative data showed that participants were positive about the intervention. Reported benefits included emotional support, meaning‐making through sharing experiences, modelling of behaviours and coping strategies, information provision and positive appraisal of well‐being through social comparison (see Box 1). Stroke survivors found the intervention was acceptable in terms of group size, venue, length of sessions, and duration of programme. Participants reported a particular strength was having a stroke survivor facilitate the group, who served as a positive role model.

Box 1 **Examples of perceived benefits among stroke survivors taking part in the intervention**

**Table:** 

*Emotional support*
I think they (the group) were very encouraging. I get a lot of encouragement, you know, and friendliness. (male stroke survivor, interview)
I enjoyed the company. They were all friendly, and they helped you. (female stroke survivor, interview)
*Meaning‐making through sharing experiences*
It gives you a chance to hear other people's experiences … You know that they can get about as well as you can and that it's not the end of the world and there's still time for improvement, that's the main thing. It is good to get out and meet people and find they can live cheerful lives. (male stroke survivor, interview)
The fear that stroke affected me disappeared. Sharing experiences dissipated the fear and made the future more meaningful. (male stroke survivor, feedback form)
*Modelling of behaviours and coping strategies*
Since I came here I get the idea this is not the end of my life. I'm going to keep on pushing. (female stroke survivor, interview)
*Information provision*
What I found very informing was the amount of things you can get (from local community services and groups). I didn't know about those things, because where I never go out from here, I don't sort of get to know about them. (female stroke survivor, interview)
*Social comparison*
Hearing how they coped so well made me realise how lucky I am compared to others. (female stroke survivor, feedback form)
I know a lot of people that have had strokes and when I look at them, you know, they're in wheelchairs or things like that and I think how lucky I am, I really say how lucky I am. (female stroke survivor, interview)

Feedback from the peer facilitator, assistant, and professionals delivering the intervention was largely positive. The facilitator identified having a second stroke survivor to assist the group was invaluable as it helped to put participants at ease and facilitated group discussions. Areas of improvement identified prior to conducting a future trial include: having a larger group to enable a broader range of experiences to be shared (assistant), and attention to potential power imbalance between invited professionals as ‘experts’ and participants by making group sessions more interactive and less focused on presentations (nurse).

## Discussion

In this paper, we have discussed the application of the revised UK MRC framework to develop a novel intervention conceptually grounded from the literature and empirical research with older stroke survivors, carers and professionals working in the delivery and commissioning of stroke and older adult services. This is a group‐based peer support intervention targeting practices to enhance resilience after stroke. A preliminary evaluation found that it was feasible to deliver the intervention and acceptable to stroke survivors receiving the programme, and peer facilitators and professionals delivering it.

Existing interventions indicate that the psychosocial consequences of stroke are not adequately addressed (Murray *et al*. 2003, National Audit Office 2010), and there is a lack of evidence of how to improve long‐term outcomes for people with stroke (Knapp *et al*. 2000, Kendall *et al*. 2007). Observational studies show that resilience is associated with improved psychosocial outcomes in older adults (Hardy *et al*. 2004, Nygren *et al*. 2005, Lamond *et al*. 2008, Netuveli *et al*. 2008, Hildon *et al*. 2009) and among people with a range of long‐term conditions (Stewart & Yuen 2011). Qualitative studies have further found that older people identify a range of psychosocial resources contributing to resilience in later life (Felten 2000, Felten & Hall 2001, Clarke & Cordman 2002, Becker & Newsom 2005, Kinsel 2005, Hildon *et al*. 2008, Windle *et al*. 2010, Wiles *et al*. 2012). However, no studies have looked at the structured promotion of resilience after stroke. Our findings highlight that resilience plays an important role in adjustment after stroke from the perspective of stroke survivors and other key stakeholders. We have shown that a range of resilience practices to promote adjustment after stroke can be targeted in a theoretically and empirically robust intervention.

### Implications for practice and future research

The findings from this study have implications for the development of interventions to promote resilience to improve psychosocial outcomes for people with stroke, which could have potential transferability to individuals with other long‐term conditions. First, this study demonstrates the utility of applying the revised UKMRC framework to systematically develop and evaluate empirically and theoretically robust interventions in health and social care. Second, engaging service users and professionals in co‐designing appropriate interventions potentially improves their relevance and impact to improve long‐term outcomes in stroke and other populations. Third, our initial assessment of feasibility of the intervention was based on a small sample of older stroke survivors. Further limitations were the low response rates and variable attendance among participants, which may introduce bias in a future trial. Prior to conducting a definite RCT of the intervention to test effectiveness (phase 3 of the UKMRC framework), a future study would entail conducting a randomised feasibility study to determine whether a full trial is feasible with a larger sample, recruited from other sources, such as General Practices, and reflecting a wider age range of people with stroke.

## Conflict of interest

No conflicts of interest have been declared.

## Source of funding

This work was funded by the Medical Research Council/Economic and Social Research Council's Life Long Health and Wellbeing programme (grant number G1001901/1).
